# Visiting crowded places during the COVID-19 pandemic. A panel study among adult Norwegians

**DOI:** 10.3389/fpubh.2022.1076090

**Published:** 2022-12-15

**Authors:** Leif Edvard Aarø, Lamprini Veneti, Øystein Vedaa, Otto R. F. Smith, Birgitte Freiesleben De Blasio, Bjarne Robberstad

**Affiliations:** ^1^Department of Health Promotion and Development, University of Bergen, Bergen, Norway; ^2^Department of Health Promotion, Norwegian Institute of Public Health, Bergen, Norway; ^3^Department of Infection Control and Preparedness, Norwegian Institute of Public Health, Oslo, Norway; ^4^Department of Psychosocial Science, University of Bergen, Bergen, Norway; ^5^Centre for Evaluation of Public Health Measures, Norwegian Institute of Public Health, Oslo, Norway; ^6^Department of Teacher Education, NLA University College, Bergen, Norway; ^7^Department of Method Development and Analytics, Norwegian Institute of Public Health, Oslo, Norway; ^8^Department of Biostatistics, Institute of Basic Medical Science, University of Oslo, Oslo, Norway; ^9^Section for Ethics and Health Economics, Department of Global Public Health and Primary Care, University of Bergen, Bergen, Norway

**Keywords:** COVID-19, panel study, cross-lagged models, crowded places, response effectiveness, self-efficacy, vulnerability, barriers

## Abstract

Non-pharmaceutical interventions, including promotion of social distancing, have been applied extensively in managing the COVID-19 pandemic. Understanding cognitive and psychological factors regulating precautionary behavior is important for future management. The present study examines the importance of selected factors as predictors of having visited or intended to visit crowded places. Six online questionnaire-based waves of data collection were conducted in April–October 2020 in a Norwegian panel (≥18 years). Sample size at Wave 1 was 1,400. In the present study, “Visited or intended to visit crowded places” for different types of locations were the dependent variables. Predictors included the following categories of items: Perceived response effectiveness, Self-efficacy, Vulnerability, Facilitating factors and Barriers. Data were analyzed with frequency and percentage distributions, descriptives, correlations, principal components analysis, negative binomial-, binary logistic-, and multiple linear regression, and cross-lagged panel models. Analyses of dimensionality revealed that a distinction had to be made between Grocery stores, a location visited by most, and locations visited by few (e.g., “Pub,” “Restaurants,” “Sports event”). We merged the latter set of variables into a countscore denoted as “Crowded places.” On the predictor side, 25 items were reduced to eight meanscores. Analyses of data from Wave 1 revealed a rather strong prediction of “Crowded places” and weaker associations with “Supermarket or other store for food.” Across waves, in multiple negative binomial regression models, three meanscore predictors turned out to be consistently associated with “Crowded places.” These include “Response effectiveness of individual action,” “Self-efficacy with regard to avoiding people,” and “Barriers.” In a prospective cross-lagged model, a combined Response effectiveness and Self-efficacy score (Cognition) predicted behavior (“Visited or intended to visit crowded places”) prospectively and vice versa. The results of this study suggest some potential to reduce people's visits to crowded locations during the pandemic through health education and behavior change approaches that focus on strengthening individuals' perceived response effectiveness and self-efficacy.

## Introduction

During the 2 years starting in March 2020, the fight against the COVID-19 pandemic dominated global public health action at an unprecedented scale. Before effective vaccines were developed, arguably the most important means to manage the pandemic were infection control advice/measures (1). Typically, infection control advice/measures refer to recommendations and regulations used by health authorities to reduce the spread of infection in the population. This may include individual hygiene such as hand washing, use of antibacterial fluids, and use of face masks. Further, social distancing measures may be used in special situations, such as keeping a minimum of 1-meter distance from others, avoiding public transport, home-office and homeschooling, health services partly offered through digital platforms, quarantine measures, travel restrictions, and generally avoiding large groups of people. The purpose of infection control advice/measures is to steer the population toward a type of behavior that reduces the spread of the virus, thereby reducing human suffering and death and averting overloading the healthcare system.

In Norway, the response to the COVID-19 pandemic was quick and firm. In early spring 2020 information about needed preventive measures to curb the pandemic was provided by the Ministry of Health and Care Services, the Norwegian Institute of Public Health, and the Norwegian Directorate of Health. This included information about behavioral precautions such as responsible hygiene and social distancing. TV channels, including the two most popular ones, continuously provided information about the pandemic, and the newspapers were filled with articles and news related to the pandemic. A national lockdown was introduced on March 12th, 2020. Within 3 weeks, the incidence of infected people (per 2 weeks) was markedly reduced from a moderate level to a very low level (2, 3). Contributing to this was a high level of compliance with health authorities' restrictions and recommendations. A contributing factor to the high compliance rates in Norway may have been the generally high level of trust in authorities found in Norway, and also across all the other Nordic countries (4). However, at the same time, adherence to quarantine/isolation in Norway was reported as low after the initial surge of infections faded nationwide and the most drastic physical distancing measures were eased (5). When the data collection of the present study started, the population was already well informed about the importance of social distancing, and restrictive measures to promote social distancing had already been introduced.

The present study focuses on visiting crowded places, which is one kind of behavior that represents a willingness to expose oneself to the risk of infection. This is the opposite of social distancing.

A search on Medline (November 15, 2022) shows about 314,000 publications on COVID-19 and corona since 2020. When combining the search with “behavior”, the number of publications is still as high as over 15,000. This newly published scientific literature on the COVID-19 pandemic is part of an even larger picture, which also comprises dramatic issues such as climate changes, natural resources depletion, and war, and behavioral, physical, and psychological effects of these disasters (6). A large proportion of studies on behavioral effects of the COVID-19 pandemic focus on adherence to recommendations from authorities in many countries and changes in pandemic-related behavior over time. In Norway, a high level of compliance has been reported (2). A distinction is often made between COVID-19 related hygiene (such as washing hands, using sanitizers, using face mask) and social distancing (7). Avoiding crowded places during pandemics is one important aspect of social distancing.

Since the 1950's, a large number of theories and conceptual models have been developed to explain and predict health-related behaviors. Among the most prominent ones are Social Cognitive Theory (8), the Health Belief Model (9), and the Reasoned Action Framework (Theory of Planned Behavior) (10). Most health behavior research has focused on behaviors other than those related to the risk of COVID-19 infection. Still, the relevance of the theories and conceptual models developed within health behavior research for understanding and predicting COVID-19-related behaviors is obvious.

As reported by Epton et al., adherence to physical distancing regulations during the COVID-19 pandemic has varied a lot, from 30 to 95% across studies, with considerable differences across countries and contexts (11). Beliefs, such as higher levels of trust in politics and science have been found to be positively correlated with adherence (12).

Cross-sectional studies have shown social and cognitive factors to predict social distancing or social distancing intentions. This is the case with knowledge (13), attitudes (14–18), perceived severity if infected (14), subjective norms (16–20), self-efficacy (19), and perceived behavioral control (16, 18–20). In one study, capacity (confidence in being able to do a specific behavior) was shown to significantly predict four indicators of social distancing (21). Based on analyses of free-text data from UK adults, Wright et al. concluded that concerns about the risk of COVID-19 for oneself and one's family and friends was particularly important for facilitating compliance with guidelines (22).

Gibson et al. (17) carried out a prospective panel study (two waves) with the Theory of Planned Behavior (TPB) predictors of social distancing (*n* = 507). Based on cross-sectional analyses of data from baseline, they found that the three classic TPB predictors, “Attitudes,” “Subjective Norms,” and “Perceived Behavioral Control” all predicted behavioral intentions significantly. Intentions measured at baseline predicted social distancing at follow-up.

Analyses of data from a few prospective longitudinal studies have confirmed the importance of several social-cognitive factors in predicting change in social distancing and avoiding crowds. This is the case with social- or subjective norms (23, 24), perceived risk of being infected (23), and perceived behavioral control (24). One prospective longitudinal study did not show significant prediction of social distancing from social cognitive predictors after adjusting for past behavior (25).

Based on analyses of data from three countries, the United States, U.K., and Germany, and combining survey and experimental approaches, Pfattheicher et al. found empathy for people most vulnerable to the virus to represent an emotional basis for motivation for physical distancing (26).

The methodological strengths of prospective longitudinal study designs are not always fully exploited. For instance, adjustments for behavior at earlier waves of data collections are not always conducted when examining how other factors predict behavior prospectively (17, 19).

During the first phase of the COVID-19 pandemic, United Kingdom, Belgium, and the Netherlands developed the CoMix study that was launched in March 2020. This study was inspired by the POLYOD study and (27) had as its main aim to describe the impact of COVID-19 restrictions on social contacts (28). The University of Bergen (UoB) and the Norwegian Institute of Public Health (NIPH) were invited to join this collaboration, and our group adapted the questionnaire and sampling design to the Norwegian context and conducted a 6-wave survey among Norwegian adults from April to October 2020. Additional countries later joined the CoMix project (29). The results presented in this publication are based on the Norwegian CoMix. During each survey, participants answered questions on experienced symptoms, attitudes toward intervention measures, visits and intentions to visit crowded locations during the last 7 days preceding the survey, and questions on social contacts during the day preceding the survey, and whether they were affected by distancing measures.

In the present study, we focused on analyzing data on people's visits and intentions to visit crowded locations and explored behavioral predictors. We measured five categories of predictors, which were anchored and defined in different ways in health behavior theories, using varying but related terminology:

**Perceived response effectiveness** – indicates to what extent various precautions are perceived as effective against infection.

In the Theory of Planned Behavior, the most proximal predictor of behavior is “Behavioral intentions.” Intentions are assumed to be determined by three factors, Attitudes, Subjective norms, and Perceived behavioral control. Attitudes are determined by an individual's belief about outcomes of performing the behavior and evaluation of these outcomes (10). “Perceived response effectiveness” is a concept which covers a broader range of actions which can be taken to counteract a health threat, for instance restrictive measures.

In Bandura's Social Cognitive Theory, “Outcome expectations” refer to an individual's expectation about the physical or social consequences, of action. Among the physical repercussions are health consequences. “Perceived response effectiveness is a broader” concept than “Outcome expectations” since the latter is limited to consequences of individual behavior. In the Health Belief Model other labels are used to cover similar behavioral predictors (9).

**Self-efficacy** is a central concept in Bandura's Social Cognitive Theory (8) and can be defined as a person's confidence in his or her ability to perform a behavior that produces a specific outcome.

In the Theory of Planned Behavior, a related concept is “Perceived behavioral control”, which is defined as a person's perception of how easy or difficult it is to perform the behavior of interest. While self-efficacy is about the perceived ability to perform a behavior, perceived behavioral control also includes the perceived control over performing a behavior (30).

**Perceived vulnerability**, also called perceived susceptibility, reflects an individual's belief about the risk of a health threat's occurrence or the risk of developing a health problem. This behavioral determinant is one of the core concepts in the Health Belief Model as well as in Protection Motivation Theory (9).

**Perceived barriers** can be defined as obstacles to taking action. In the context of our study, **facilitating factors** are conditions that makes it easier to follow recommended actions.

The aims of this study were (i) to examine properties of the scale used to measure “Visited or intended to visit crowded places”, (ii) to examine bivariate associations among predictors as well as between predictors and “Visited or intended to visit crowded places”, (iii) to examine the “impact” of the whole set of predictors on the “crowded” outcome variables for each wave of data collections, and (iv) to examine prospective, cross-lagged associations between selected predictors and behavior.

## Methods

### Data collections

Data collections were carried out by the market research company Ipsos. The same company was used for data collections in other European countries involved in the CoMix study. Eligible persons were recruited from existing members of the Ipsos Norwegian online panel. From this panel, a representative sample was drawn. The sample was similar to the Norwegian population with respect to age, gender, geographic location, and socio-economic status. Panel members were invited to participate by e-mail on a random day of each week of data collection. In case of non-response, up to two reminder e-mails were sent. Members who indicated that they no longer wanted to participate in the survey were replaced by other panel members of similar age and gender. In case of drop-out, additional persons were recruited in the same way.

Data were collected between April and October 2020. The first data collection (wave) took place 24th−30th April 2020, and 1400 participants were enrolled. This sample size at Wave 1, with 15% dropout at each of the consecutive waves was considered sufficient to allow us to monitor the social contact patterns in Norway over the 6-month period with adequate precision. Six waves of data collection over the 6 months were performed with a duration of 1 week per wave. In the sixth and final wave, the number of participants was 645. Since some recruitment of new participants took place before the second, fourth and fifth data collections, the total number of participants who filled out the questionnaire in at least one of the data collections was 1,718. A complete overview of recruitment to the study is shown in Supplementary Figure S1.

Participants received “panelist points” that could be exchanged for shopping vouchers for each questionnaire they filled out. The questionnaires were in Norwegian, and data were collected using an online webform developed by the CoMix consortium. The study was carried out by the University of Bergen and the Norwegian Institute of Public Health (Norway) in collaboration with the London School of Hygiene and Tropical Medicine (LSHTM), United Kingdom, and the University of Antwerp, Belgium.

### Questionnaire

Panel members were asked questions about demographics, social contact behavior, personal preventive measures, and exposure to and impact of different social distancing interventions.

The outcome variables in the present study are based on a scale with nine items regarding visits to or intentions to visit crowded places. The wording of one of these questions and the response categories are shown in Table 1. The venues covered were: (i) Pub, bar, or café, (ii) Restaurant, (iii) Cinema, (iv) Supermarket or other store for food, (v) Sports event (as a spectator), for instance football match, (vi) Sports event (as an athlete), practicing sessions included, (vii) Religious gathering, (viii) Indoor location with more than 100 people present, (ix) Outdoor location, with more than 100 people present.

**Table 1:** Visited or intended to visit supermarket or other store for food during the last 7 days for each wave of data collections.

**Did you visit or intend to visit any of the following events or locations in the last 7 days? Supermarket or other store for food**	**Wave 1**		**Wave 2**		**Wave 3**		**Wave 4**		**Wave 5**		**Wave 6**	
	***N***	**%**	***N***	**%**	***N***	**%**	***N***	**%**	***N***	**%**	***N***	**%**
Yes, I visited this event or location*	1,180	84.3	1,024	86.6	851	84.1	809	86.9	664	86.5	540	83.7
No, I planned to visit, but it was canceled because of the COVID-19 epidemic*	23	1.6	16	1.4	18	1.8	23	2.5	9	1.2	9	1.4
No, I planned to visit but chose not to go because of the COVID-19 epidemic	54	3.9	32	2.7	28	2.8	20	2.1	17	2.2	14	2.2
I intended to visit, but I had to cancel for reasons unrelated to the COVID-19 epidemic*	11	0.8	11	0.9	15	1.5	8	0.9	7	0.9	9	1.4
No, I did not visit and had no plans to visit this kind of events or locations	132	9.4	99	8.4	100	9.9	71	7.6	71	9.2	73	11,3
Total	1,400	100.0	1,182	100.0	1,012	100.0	931	100.0	768	100.0	645	100.0

Frequency and percentage distributions.

^*^ When this variable was dichotomized, these three categories indicated by asterisk were combined into one group (code 1) (visited or planned/intended to visit), leaving the remaining two categories in a second group (code 0) (did not visit; planned to visit, but chose not to).

The predictors used in the present study included measures of (i) Vulnerability – 3 items, (ii) Response effectiveness – 11 items, (iii) Self-efficacy – 7 items, (iv) Facilitating factors – 3 items, and (v) Barriers – 2 items. The wording of these items is shown in **Table 3**.

### “Visiting crowded places” variables

As explained above, the questionnaire listed nine types of “crowded places” which could have been visited. There were five response categories for each (Table 1). This measurement was repeated in all six waves of data collection, providing a total of 270 response possibilities for each study participant.

For each item and for each wave, the five response categories were dichotomized. The two responses which meant that they did not visit or canceled due to the COVID-19 pandemic were coded as 0 (zero). The three response categories which meant that they visited or intended/planned to visit and did not cancel themselves were coded as 1 (one) (see Table 1).

Principal components analyses of the nine dichotomies measured at baseline confirmed that eight of the nine items were highly correlated and could be combined into one countscore denoted “Visiting or intended to visit crowded places.” These were all venues that were visited by rather few. The Cronbach's alpha value for the “Crowded places” group of dichotomies was 0.842 at Wave 1. The alpha value decreased when “Supermarket or other shop for food or groceries” was included in the scale.

The only item which was not included in the countscore was “Visited or intended to visit supermarket or other store for food.” This venue was visited by a large proportion of the study participants at each data collection and was analyzed separately as a single dichotomous outcome.

This means that we ended up with two outcome variables, one countscore varying from 0 to 8 called “Visited or intended to visit crowded places” (or shorter: “Crowded places”) and one dichotomous variable coded 1 for “Visited or intended to visit supermarket or other store for food,” and 0 otherwise. These two outcome variables could be analyzed across all six waves of data collections.

### Statistical analyses

Data were analyzed with SPSS version 27 and Mplus version 8.7. Data were described with one-way frequency and percentage distributions and basic descriptive statistics for metric variables (means, standard deviations, skewness). The dimensionality of scales was examined with Principal Components Analysis. Associations between count outcomes and metric predictors (meanscores) were analyzed using negative binomial regression models with log link, and associations between binary outcomes and metric predictors using binary logistic regression. The same set of analyses were repeated with multiple linear regression, to estimate multiple *R*^2^. For the analysis of longitudinal prediction across all six waves, we used cross-lagged panel models with maximum likelihood robust estimation (MLR).

In addition to testing simple cross-lagged models, we also used Random Intercept Cross-Lagged Models (RI-CLPM) and Latent Curve Models with Structured Residuals (LCM-SR) (31). Both methods explicitly model stable between-person variance and allow for the examination of autoregressive and cross-lagged effects at the within-person level. This is in contrast to the traditional Cross-Lagged Panel Model, which does not distinguish between- and within-person effects. Both models also implicitly account for unmeasured time-invariant confounders under the assumption that the influence of these confounders is constant across measurement occasions. Additionally, the LCM-SR accounts for influences of unmeasured time-varying confounders that change linearly over time. The interpretation of within-person parameters is similar for both models. The autoregressive parameters provide information about the within-person stability of the variable of interest, which is different from the interpretation in traditional CLPM in the sense that the latter provides information about the stability of the relative position of individuals. The cross-lagged paths indicate the degree to which the deviation from the person-mean in variable X at time T is associated with the deviation from the person-mean in variable Y at time T+1 while controlling for the previous deviation from the person-mean in variable B, and vice versa (31).

Instead of sumscores, we used meanscores (sum of scores divided by the number of items). This allows us to preserve the metrics of the original scales. Meanscores for a scale were produced for cases with valid responses to at least half of the items.

During the analyses of data, several decisions had to be made. When analyzing the outcome variables (“Crowded places”), not all items could be combined into one count variable. Based on Principal Components Analyses we decided that one item stood out from others and had to be analyzed separately as a single item: “Visited supermarket or other store for food.” All other “Crowded places” indicators could be combined into one countscore, which turned out to also obtain a high Cronbach's alpha value. Also, based on Principal Components analyses, some of the groups of predictors were split into subgroups. Three single items were excluded from meanscores because they had a low number of valid observations, or they showed unexpected directions of associations (based on bivariate analyses). Based on regression analyses of data from baseline, we decided to drop the “Visited supermarket or other store for food” from further analyses of prediction of behavior and focus on the “Crowded places” countscore exclusively.

Based on multiple regression analyses with “Crowded places” as the outcome variable, for each wave separately, we decided to limit the final cross-lagged model analyses to include only four sets of variables: “Crowded places,” “Barriers,” “Effectiveness of taking individual action,” and “Self-efficacy with regard to avoiding people.” After testing several alternative cross-lagged models, a rather simple one including two cognitive predictors combined (“Effectiveness of taking individual action” and “Self-efficacy with regard to avoiding people”) and the countscore for “Crowded places” was chosen.

## Results

### Individual items and meanscore descriptives

As shown in Table 1, for all waves of data collection, most respondents had visited supermarket or other store for food during the last 7 days (between 83.7 and 86.9%). The proportions who had either visited or planned/intended to visit, but canceled for external reasons, were slightly higher (between 86.5 and 90.3%).

For the remaining eight outcome variables, distributions across all waves are not presented individually. Instead, we show percentage distributions of the count variable (Table 2). The proportions who had not visited or decided not to visit any of the locations varied between 52.1% (Wave 4) and 68.7% (Wave 1). All distributions were highly right-skewed.

**Table 2:** Number of types of crowded locations visited—or was intended/planned to visit—during the last 7 days, for each wave of data collections.

**Number of types of locations**	**Wave 1**		**Wave 2**		**Wave 3**		**Wave 4**		**Wave 5**		**Wave 6**	
	***N***	**%**	***N***	**%**	***N***	**%**	***N***	**%**	***N***	**%**	***N***	**%**
0	962	68.7	713	60.3	595	58.8	485	52.1	446	58.1	406	62.9
1	210	15.0	224	19.0	238	23.5	228	24.5	192	25.0	31	20.3
2	85	6.1	139	11.8	100	9.9	139	14.9	85	11.1	66	10.2
3	47	3.4	46	3.9	28	2.8	31	3.3	21	2.7	19	2.9
4	28	2.0	19	1.6	13	1.3	10	1.1	5	0.7	9	1.4
5	25	1.8	15	1.3	8	0.8	8	0.9	1	0.1	3	0.5
6	7	0.5	6	0.5	5	0.5	3	0.3	3	0.4	0	0.0
7	5	0.4	5	0.4	25	2.0	27	2.9	15	2.0	11	1.7
8	31	2.2	15	1.3	0	0.0	0	0.0	0	0.0	0	0.0
Total	1,400	100.0	1,182	100.0	1,012	100.0	931	100.0	768	100.0	645	100.0

Supermarket or other store for food excluded. Frequency and percentage distributions.

All behavioral predictors' items had scales of 1 (strongly disagree/not at all)-5 (strongly agree/very), or 1 (not at all)-−4 (very). For the 5-level items, the mean scores ranged from 2.21/5 (facilitating barriers) to 4.42/5 (perceived vulnerability. For the 4-level items (Response effectiveness), the mean scores generally were a bit higher and ranged between 3.07/4 and 3.62/4 (Table 3). Since all items belong to distinct categories of predictors, they are presented groupwise with alpha values and descriptives (means and standard deviations) for the meanscores. Each group was analyzed in a series of principal component analyses (tables not shown). This led to the division of some groups into subgroups.

**Table 3:** Single item and meanscore descriptives at Wave 1.

**Scale/range**	**Items**	**Mean**	**S.D**.	***N***	**Cronbach's alpha**	**Mean**	**S.D**.	***N***
Response effectiveness - Individual action 1 = Not at all effective 4 = Very effective	Reducing the number of people you meet	3.53	0.61	1,378	0.78	3.52	0.49	1,385
	Staying home for 7 days if mild symptoms	3.33	0.69	1,324				
	Staying home for 7 days if more severe symptoms	3.61	0.62	1,368				
	Avoiding crowded places	3.62	0.57	1,379				
- Individual action if someone in household has symptoms 1 = Not at all effective 4 = Very effective	Staying home for at least 14 days if others in household had mild symptoms such as mild cough	3.22	0.76	1,314	0.76	3.37	0.63	1,363
	Staying home for at least 14 days if others in household had severe symptoms	3.52	0.63	1,349				
- Restrictive measures 1 = Not at all effective 4 = Very effective	Closing schools	3.08	0.77	1,309	0.83	3.32	0.53	1,378
	Closing bars, restaurants, cinemas, etc.	3.47	0.65	1,372				
	Banning the use of public transport	3.40	0.63	1,377				
	Banning international travel into this country	3.56	0.66	1,365				
	Banning travel within this country	3.07	0.75	1,346				
Self-efficacy - Avoid meeting other people 1 = Not at all confident 5 = Very confident	Confident you could reduce the number of people you meet	3.54	0.66	1,381	0.73	3.51	0.57	1,387
	Confident you could avoid crowded places	3.55	0.64	1,388				
	Not use public transport	3.43	0.82	1,375				
- Stay home 1 = Not at all confident 5 = Very confident	Confident you could stay home for 7 days if you had mild symptoms	3.35	0.77	1,368	0.84	3.42	0.62	1,387
	Confident you could stay home for 7 days if you had more severe symptoms	3.67	0.60	1,388				
	Confident you could stay home for 14 days if others in household had mild symptoms	3.21	0.85	1,345				
	Confident you could stay home for 14 days if others in household had more severe symptoms	3.44	0.73	1,359				
Vulnerability (own or others') 1 = Strongly disagree 5 = Strongly agree	Coronavirus would be a serious illness for me	3.17	1.35	1,253	0.38	3.51	0.84	1,346
	I am likely to catch coronavirus	2.82	1.15	1,257				
	If I don't follow government's advice, I might spread the virus to others	4.42	1.05	1,366				
Facilitating factors 1 = Strongly disagree 5 = Strongly agree	If I could not work because of coronavirus, I would still get paid	4.14	1.28	808	0.38	3.85	0.99	870
	If I had to isolate myself for 7 days because of coronavirus, someone else would be able to look after my children	3.42	1.46	326				
	I have enough food and supplies to last for 7 days, if I had to isolate myself	3.79	1.36	1,380				
Barriers 1 = Strongly disagree 5 = Strongly agree	Other people expect me to work, even when I am ill	2.21	1.33	829	0.43	2.25	1.17	1,375
	If I had to isolate myself for 7 days, this would cause problems for other people who I don't know	2.36	1.36	1,357				

Reflective measurement models can be assumed for those scales or subscales that can be seen as reflecting underlying cognitive properties (vulnerability, response effectiveness, and self-efficacy). For most of these scales, the alpha values at Wave 1 varied from 0.73 to 0.84, indicating good reliability. There was, however, one exception. The alpha value for the Vulnerability scale was low at 0.38.

The number of valid observations for one of the Facilitating factors items about isolation was very low (*n* = 326), and this variable was therefore excluded from subsequent analyses.

### Bivariate associations with “visited supermarket or other store for food”

Bivariate associations between all the candidate predictors (25 single items and 8 meanscores) and our two behavioral outcome variables (Visited or intended to visit supermarket or other store for food; Visited or intended to visit crowded locations) are presented in Table 4.

**Table 4:** Behavioral outcomes by cognitive predictors and facilitating factors/barriers. Bivariate associations.

		**A: Bivariate coefficients Number of types of locations visited**	***N***	**B: Bivariate coefficients Visited supermarket or other store for food**	***N***
Response effectiveness	Meanscore	−0.584*** (−0.733 /−0.436)	1,385	1.126 (0.825–1.535)	1,385
– individual action	How effective: reducing the number of people you meet	−0.398*** (−0.521 /−0.275)	1,378	1.187 (0.927–1.521)	1,378
	How effective: staying home for 7 days if you have a mild symptom such as a mild cough	−0.217*** (−0.331 /−0.102)	1,324	0.875 (0.689–1.112)	1,324
	How effective: staying home for 7 days if you have more severe symptoms such as severe cough or a high temp	−0.474*** (−0.591 /−0.356)	1,368	1.245 (0.985–1.574)	1,368
	How effective: avoiding crowded places	−0.447*** (−0.573 /−0.322)	1,379	1.113 (0.854–1.451)	1,379
Response effectiveness	Meanscore	−0.240*** (−0.363 /−0.117)	1 363	0.735* (0.563–0.959)	1,363
– Individual action if someone in household	Staying home for at least 14 days if others in household has mild symptoms	−0.101 (−0.207/0.004)	1,314	0.725** (0.578–0.909)	1,314
has symptoms	Staying home for at least 14 days if others in household has severe symptoms	−0.303*** (−0.424/−0.182)	1,349	0.897 (0.695–1.159)	1,349
Response effectiveness	Meanscore	−0.481*** (−0.623/−0.338)	1,378	0.844 (0.624–1.141)	1,378
– Restrictive measures	Closing schools	−0.180*** (−0.283/−0.076)	1,309	0.747** (0.600–0.930)	1,309
	Closing bars, restaurants, cinemas, etc.	−0.461*** (−0.575/−0.346)	1,372	1.057 (0.839–1.331)	1,372
	Banning the use of public transport	−0.396*** (−0.516/−0.275)	1,377	1.023 (0.799–1.309)	1,377
	Banning international travel into this country	−0.261*** (−0.375/−0.147)	1,365	1.021 (0.807–1.291)	1,365
	Banning travel within this country	−0.215*** (−0.319/−0.111)	1,346	0.812 (0.655–1.008)	1,346
Self-efficacy	Meanscore	−0.584*** (−0.716/−0.451)	1,387	0.803 (0.601–1.073)	1,387
- avoid meeting other people	Confident you could reduce the number of people you meet	−0.420*** (−0.534/−0.306)	1,381	0.877 (0.683–1.126)	1,381
	Confident you could avoid crowded places	−0.403*** (−0.520/−0.286)	1,388	0.786 (0.601–1.027)	1,388
	Not use public transport	−0.361*** (−0.455/−0.266)	1,375	0.935 (0.768–1.139)	1,375
Self-efficacy	Meanscore	−0.402*** (−0.524/−0.280)	1,387	0.731* (0.556–0.962)	1,387
- stay home	Confident you could stay home for 7 days if you had mild symptoms	−0.275*** (−0.376/−0.175)	1,368	0.712** (0.565–0.896)	1,368
	Confident you could stay home for 7 days if you had more severe symptoms	−0.374*** (−0.488/−0.259)	1,388	1.079 (0.840–1.386)	1,388
	Confident you could stay home for 14 days if others in household had mild symptoms	−0.155*** (−0.248/−0.062)	1,345	0.731** (0.595–0.897)	1,345
	Confident you could stay home for 14 days if others in household had more severe symptoms	−0.338*** (−0.440/−0.237)	1,359	0.871 (0.696–1.090)	1,359
Vulnerability	Meanscore	−0.015 (−0.109/0.080)	1,346	1.036 (0.858–1.036)	1,346
	Coronavirus would be a serious illness for me	0.005 (−0.056/0.066)	1,253	0.820** (0.723–0.930)	1,253
	I am likely to catch coronavirus	0.129*** (0.058/0.201)	1,257	1.075 (0.932–1.240)	1,257
	If I don't follow government's advice, I might spread the virus to others	−0.133*** (−0.207 /−0.059)	1,366	1.277** (1.124–1.452)	1,366
Facilitating factors	Meanscore	−0.170*** (−0.268 /−0.072)	870	0.896 (0.734–1.094)	870
	If I could not work because of coronavirus, I would still get paid	−0.087* (−0.166 /−0.008)	808	1.036 (0.887–1.208)	808
	If I had to isolate myself for 7 days because of coronavirus, someone else would be able to look after my children	0.057 (−0.051/0.164)	326	0.862 (0.697–1.067)	326
	I have enough food and supplies to last for 7 days, if I had to isolate myself	−0.138*** (−0.196/−0.080)	1,380	0.863* (0.763–0.977)	1,380
Barriers	Meanscore	0.255*** (0.189/0.322)	1,375	0.901 (0.790–1.026)	1,375
- barriers	Other people expect me to work, even when I am ill	0.167*** (−0.094/0.240)	829	0.806** (0.695–0.934)	829
	If I had to isolate myself for 7 days, this would cause problems for other people who I don't know	0.193*** (0.135/0.251)	1,357	0.968 (0.863–1.085)	1,357

^*^p < 0.05; ^**^p < 0.01; ^***^p < 0.001.

A: Number of kinds of locations visited (or intended to visit) by cognitive predictors and facilitating factors and barriers at Wave 1. Bivariate associations. Negative binomial regression with loglink for count outcomes.

B: Visited or intended to visit Supermarket or other store for food by cognitive predictors and facilitating factors and barriers at Wave 1. Bivariate associations. Binary logistic regression analysis.

Out of 25 single item associations with “Visited supermarket or other store for food”, only six turned out to be statistically significant and with odds ratio values on the expected side of 1.00. For predictors assumed to protect against visiting crowded places, odds ratio values lower than 1.00 are expected. For predictors assumed to increase the risk of visiting crowded places, odds ratio values higher than 1.00 are expected. The odds of having visited or intended to visit “Supermarket or other store for food” was significantly under 1.00 for (i) “Coronavirus would be a serious illness for me,” (ii) effectiveness of “staying home for at least 14 days if others in household had mild symptoms such as mild cough,” (iii), effectiveness of closing schools, (iv) “Confident could stay home for 7 days if mild symptoms,” (v) “Confident could stay home for 14 days if others in household had mild symptoms,” and (vi) “Had enough food and supplies for at least 7 days if had to isolate.” Keeping in mind that all predictors had 1–4 or 1–5 scale ranges, the odds ratio values varied between 0.712 and 0.863, which means moderate to rather weak associations.

Two significant associations with “Supermarket or other store for food” were in the unexpected direction: “If I don't follow the government's advice, I might spread the virus to others” (O.R. = 1.277) and “Other people expect me to work, even when I am ill” (O.R. = 0.806).

Among the 8 meanscores, only two turned out to have a significant association with the visited or intended to visit “Supermarket or other shop for food” dichotomy, namely “Individual action response effectiveness if someone in household has symptoms” (O.R. = 0.735) and “Self-efficacy with regard to staying home” (O.R. = 0.731).

### Bivariate associations with “visited or intended to visit crowded places” (number of locations)

Out of 25 single item associations with “Crowded places,” 23 were significant (Table 4). All these 23 coefficients except one were in the expected direction. Associations with protective factors were negative, while associations with barriers were positive. Coefficients varied from −0.087 to−0.474 (barriers: 0.167 and 0.193), which in this context means weak to medium strong associations.

One significant association with “Crowded places” was in the unexpected direction: “I am likely to catch the coronavirus” (Coefficient = 0.129).

Among the eight associations between meanscores and “Crowded places,” seven turned out to be significant, and all were in the expected direction. The non-significant one was the meanscore for Vulnerability. This could be expected since only one of the three items used to produce this meanscore was significant in the expected direction and one item was significant in the “wrong” direction (as described above). The associations with the significant meanscores varied from−0.170 to−0.584 (Barriers: 0.255). The strongest associations were found for “Response effectiveness of individual action” and “Self-efficacy regarding avoiding meeting other people (both coefficients = 0.584).

### Associations between predictors

Since associations between predictors and behavioral outcome were less consistent, and in most cases non-significant with the “Supermarket or other store for food” dichotomous outcome, we decided to produce a set of meanscores which would first of all serve the purpose of predicting the “Crowded places” outcome variable.

A couple of modifications of meanscore composition were deemed necessary. For the vulnerability domain, only the third item was used (“If I don't follow government's advice, I might spread the virus to others”). This is a rather exceptional item to be used in the context of vulnerability, since it is not about own, but rather others' vulnerability. This single item may therefore rather be labeled “Risk of spreading the COVID-19 virus to others.”

Concerning the “Facilitating factors” meanscore, the second item (“If I had to isolate myself for 7 days because of coronavirus, someone else would be able to look after my children”) was removed because it had few valid answers (*n* = 326). This was obviously the case because it was only relevant for parents with small children.

Means and standard deviations of meanscores, as well as number of missing among those who participated at each data collection were inspected (Supplementary Table S2). Among the participants in each wave of data collections, the proportion of missing answers was generally quite low.

Correlations between meanscores at Wave 1 are shown in Table 5. All correlations between response effectiveness and self-efficacy meanscores were relatively high, from 0.410 to 0.608. “Risk of infecting others” and “Facilitating factors” were significantly correlated with all the response effectiveness and self-efficacy meanscores, but correlations were lower (from 0.135 to 0.362). “Barriers” was negatively correlated with all other variables (five significant correlations) with correlations from −0.063 to −0.138.

**Table 5:** Cognitive factors and facilitators and barriers – intercorrelations at Wave 1 (*n* = 1,400).

	**If I don't follow government's advice, I might spread the virus to others (Single item, one out of three under “Vulnerability”)**	**Response effectiveness – Individual action**	**Response effectiveness – Individual action if someone in household has symptoms**	**Response effectiveness - Restrictive measures**	**Self-efficacy – Avoiding people**	**Self-efficacy – Stay home**	**Facilitating factors ¤ (Two out of three items)**
Response effectiveness – Individual action	0.362***						
Response effectiveness – Individual action if someone in household has symptoms	0.185***	0.552***					
Response effectiveness - Restrictive measures	0.270***	0.608***	0.448***				
Self-efficacy – Avoiding people	0.208***	0.478***	0.350***	0.468***			
Self-efficacy – Stay home	0.192***	0.482***	0.518***	0.410***	0.558***		
Facilitating factors	0.080***	0.207***	0.135***	0.170***	0.260***	0.218***	
Barriers	−0.083**	−0.063*	−0.075**	−0.043	−0.082**	−0.138***	−0.080

^*^p < 0.05; ^**^p < 0.01; ^***^p < 0.001. ¤ If I could not work because of coronavirus, I would still get paid; I have enough food and supplies to last for 7 days, if I had to isolate myself.

### Analyses across waves

Table 6 shows a series of multiple negative binomial regression analyses with “Visited or intended to visit crowded places” as the outcome variable and all the meanscores described above as predictors. To improve interpretability, all predictors were standardized, and the few missing observations in each data collection were replaced with means. Since there were high intercorrelations between predictors, it was expected that the strongest predictors would suppress those which were less strongly associated with the outcomes, which turned out to be correct. Three meanscores dominated across the six waves: “The effectiveness of taking individual action,” “Self-efficacy with regard to avoiding people,” and “Barriers.” All coefficients with “Self-efficacy with regard to avoiding people” and “Barriers” were significant and in the expected direction. For “Response effectiveness of individual action,” all coefficients were in the expected direction, and three of them were significant.

**Table 6:** Visited crowded places by cognitive predictors and facilitating factors and barriers.

		**Wave 1 (*n* = 1,400)**	**Wave 2 (*n* = 1,182)**	**Wave 3 (*n* = 1,012)**	**Wave 4 (*n* = 931)**	**Wave 5 (*n* = 768)**	**Wave 6 (*n* = 645)**
If I don't follow government's advice, I might spread the virus to others (single item)		0.034	−0.039	−0.047	−0.101	−0.102	0.086
Response effectiveness	Taking individual action	−0.159**	−0.065	−0.153*	−0.160*	−0.135	−0.150
	Taking individual action if someone in household has symptoms	0.060	−0.102	0.073	−0.072	0.052	−0.141
	Restrictive measures	−0.058	0.046	0.027	0.153*	−0.125	0.015
Self-efficacy	Avoiding people	−0.227***	−0.200***	−0.170**	−0.137*	−0.137*	−0.114
	Staying home	−0.010	0.093	0.061	0.069	0.120	0.160
Facilitating factors¤		−0.047	−0.091*	−0.121*	−0.057	−0.030	−0.084
Barriers		0.274***	0.150***	0.160***	0.212***	0.215***	0.197**

Multiple regression models (Negative binomial distribution with log link for count outcomes), one for each Wave. Standardized predictors. Two out of three items: If I could not work because of coronavirus, I would still get paid; I have enough food and supplies to last for 7 days, if I had to isolate myself.

*p < 0.05; **p < 0.01; ***p < 0.001.

To obtain estimates for overall prediction, multiple linear regression analyses were carried out, one for each wave, similar to the ones carried out with negative binomial regression. The amount of explained variance varied between 20 and 25% across the six waves.

The multiple binary logistic regression analyses which were carried out for each wave, with visited or intended to visit “Supermarket or other store for food” as the dependent variable, showed Cox & Snell pseudo R2 varying from 0.021 to 0.057, corresponding to between 2.1 and 5.7% explained variance in the dependent variable (Supplementary Table S3).

### Longitudinal modeling

In order to prepare for the longitudinal modeling, a meanscore called “Cognition” was constructed based on the two cognitive factors which had proven strongly and consistently associated with “Crowded places” (“The effectiveness of taking individual action” and “Self-efficacy with regard to avoiding people”).

When analyzing interactions between “Cognition” and “Barriers,” both variables were standardized, and a product variable was constructed. Extreme values on the product variable (outliers) were truncated to scores corresponding to plus or minus 3 standard deviations. All predictor variables (cognition, barriers, interaction variable) as well as “Crowded” were transferred from SPSS to Mplus for longitudinal modeling.

**Figure 1:**
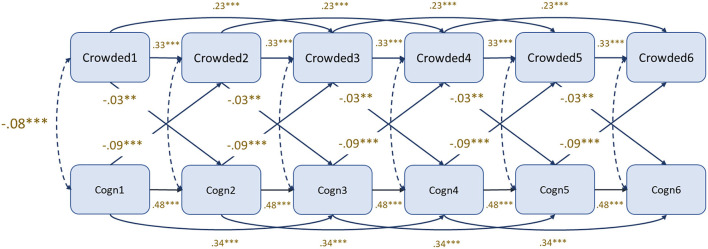
Cognitive factors (“Response effectiveness of individual action” and “Self-efficacy with regard to avoiding people”) and behavior (crowded places). Cross-lagged panel model. Maximum likelihood robust estimator (MLR). χ^2^ = 209.428; D.f. = 59; *P* < 0.001; RMSEA = 0.039 (0.033–0.044); CFI = 0.924; TIL = 0.916.

Figure 1 shows a cross-lagged panel model with Cognition and Behavior (Crowded places) included. A double set of autoregression coefficients (T1–T2, T2–T3, etc. as well as T1–T3, T2–T4, etc.) had to be applied to obtain a good model fit. Each set of autoregression coefficients (behavior → behavior; cognition → cognition) were constrained to be equal across time. All wave-specific correlations were constrained to be equal. And cross-lagged coefficients were set equal for each direction (behavior → cognition; cognition → behavior).

The model achieved good fit statistics [RMSEA = 0.039 (0.033–0.044); CFI = 0.924; TIL = 0.916]. All autoregression coefficients were significant (*p* < 0.001) and stronger for cognition (0.48 and 0.34) than for behavior (crowded places) (0.33 and 0.23).

As expected, associations between cognition and behavior within waves were negative (coeff = −0.08). The higher scores on the cognitive factor, the lower scores on the behavior factor (less inclined to visit crowded places). There were also significant negative cross-lagged associations (behavior → cognition: −0.03; cognition → behavior: −0.09).

Multigroup analyses were carried out for gender. A model where all coefficients were constrained as described above (a nested model) was tested against a model where cross-correlations were allowed to be different for men and women but constrained to be equal for each causal direction within gender (a comparison model). The Satorra-Bentler scaled chi-square test of differences was applied. No significance was obtained (χ^2^ = 3.13831; D.F. = 2; *p* > 0.05).

An extended cross-lagged panel model with “Barriers” added did not lead to any increase in explained variance (R2) in “Crowded places” in any of the outcome waves (waves 2–6). A second extension was done by adding interaction terms between “Cognition” and “Barriers” for each of the relevant predictor waves (waves 1–5). Again, no increase in R2 for “Crowded” in any of the waves (waves 2–6) was observed. And finally, random intercept and random intercept and slope models were tested (15). No significant cross-lagged associations were obtained in these models. This can be interpreted as a confirmation that the cross-lagged effects revealed in the model shown in Figure 1 are mainly caused by between-person and not within-person variation.

## Discussion

Analyses of dimensionality and inspection of distributions confirmed a split between visited or intended to visit “Supermarket or other store for food” on one hand and the other eight items representing “Crowded places” on the other. The former was highly prevalent, while visits to the other “Crowded places” were relatively few. This discrepancy probably reflects that even in times of pandemics, grocery shopping is hard to avoid for most adults. Going to restaurants, football matches or attending religious gatherings is to a larger extent determined by each individual's preferences. This is consistent with the notion that the degree to which contextual factors determine behavior, like visiting crowded places, varies (32).

Decisions about visiting crowded places were probably, during the COVID-19 pandemic, to a large extent under the influence of external factors, restrictive measures being the most visible and important. An overview of control measures implemented during each data collection occasion is shown in Supplementary Table S2. Although the purpose of the present study is not to link behavioral precautions to specific measures, the multitude of measures constitutes an essential context for variation in the behaviors studied. The stronger the contextual influences, the weaker the personal decision processes will function in controlling behavior.

The eight “Crowded” items we used for constructing a count-score (number of types of crowded places visited) proved to have high internal consistency. High internal consistency was also found for five of the six cognitive scales and subscales to be used as predictors in subsequent statistical analyses and is consistent with previous studies [see for instance reference (33)].

One of the “Vulnerability” items was about the risk of infecting others, not the personal risk of being infected. However, we decided to include this item in subsequent analyses since it was significantly associated with “Crowded places” and in the expected direction. Avoiding infecting others, or potentially avoiding stigma associated with infecting others, appeared to serve as a motive for eluding crowded places.

As previously noted, two significant associations with “Supermarket or other store for food” were in the unexpected direction. One was for the variable expressing perceived risk of spreading the coronavirus to others. Rather than functioning as a measure of vulnerability, in which case high perceived risk would be expected to be associated with fewer visits to supermarkets, this item may have reflected that those who visited crowded locations realized that they were more exposed and had an increased risk of infecting others. The other one was “Other people expect me to work, even when I am ill.” The relevance of this predictor for predicting visits to a location like “Supermarket or other store for food” may be questioned.

As also reported above, there was one significant association in the unexpected direction in the bivariate analyses with “Crowded places” as the dependent variable. Again, this was also for a “Vulnerability” item: “I am likely to catch the coronavirus.” A similar explanation is possible, that rather than functioning as a measure of vulnerability, this item may reflect that those who visited crowded locations perceived themselves at a higher risk of being infected.

None of these three items with significant associations in the unexpected direction was included in the final cross-lagged models presented in this paper, and therefore they do not influence the results from these analyses.

The proportion of significances and the consistency of associations were much higher for the “Crowded places” sumscore than for the single item outcome visited or intended to visit “Supermarket or other store for food.” This finding may have to do with the fact that the former is a multi-item outcome (countscore), while the latter is a single item outcome. Countscores based on multiple, positively correlated items tend to have higher reliability than single items. Generally, high reliability means stronger associations with correlates.

Behavior is sometimes determined mainly by contextual factors and less so by individuals' preferences and decisions. Going to supermarkets or other stores for food may to a large extent be under contextual control. Buying food and other necessities is necessary even during pandemics. One may also alter behavior by shopping at different times during the day. Less strong associations with potential predictors were therefore expected.

The importance of the cognitive factors in predicting other types of “Crowded places” also shows up in the multiple regression analyses which were carried out for each wave. The same predictors were not equally successful as predictors of the single item outcome. Crowded places other than supermarkets and stores for food were to varying extent influenced by lockdown and other restrictive public health measures across the six waves, and these contextual factors are likely to have at least partially diluted the predictive power of individuals' personal preferences.

An important finding in this study is the dominance of three factors over the other factors when predicting “Crowded places” in multiple regression models. The strongest and most consistent ones were “Response effectiveness of individual action,” “Self-efficacy with regard to avoiding people,” and “Barriers.” This result is not surprising. Research based on several social-cognitive models for explaining health-related behavior support this finding. Health behaviors (or behavioral intentions) are predominantly determined by what people believe will be the consequences of performing a specific behavior, their degree of conviction that they can carry out this behavior, and the presence or absence of external barriers.

Other relevant concepts could have been measured. For instance, if the study had been guided by the Theory of Planned Behavior (TPB), it would have been necessary to include items aimed at measuring subjective norms. Since the 2010 version of the TPB, a distinction is made between descriptive norms (the behavior of significant others like family, friends, or colleagues) and injunctive norms (what significant others expect). Another important factor, which in the TPB functions as a mediator between the model's main predictors (attitudes, subjective norms, and perceived behavioral control) and behavior, is “Intentions.” Furthermore, additional aspects of the behavioral predictors included in this study would have to be considered for inclusion, and the TPB guidelines for how to measure the best possible way might have proven useful (34, 35). These are just some of the changes that could have contributed to improving both the cross-sectional and the longitudinal prediction of behavior.

Most previous studies of predictors of outcome variables such as “Visited or intended to visit crowded places” are cross-sectional. Prospective longitudinal studies are few, but important to throw at least some more light on causality. Since the present study measured behavior and behavioral predictors in the same way and in the same sample across six waves of data collections, data from this study are well suited for examining cross-lagged associations. Of particular importance is to determine to what extent cognitive factors predict behavior over time. In our data, “Cognition” (a combination of Response effectiveness of individual action and Self-efficacy with regard to avoiding other people) turned out to predict “Crowded places” significantly. Behavior was also found to predict cognition prospectively. This finding is consistent with Bandura's ideas of “Reciprocal determinism” (8). In our context it is imperative to understand to what extent cognitive factors predict behavior prospectively. Health education and health information approaches to influencing health behavior rest on the assumption that changes in cognition will have behavioral consequences.

The conclusion that can be drawn from the cross-lagged model is that scores on one factor predicts changes in scores on the other factor prospectively. The associations were, however, rather small. One step closer to demonstrating causality would be to present results based on a random intercept (or random intercept and slope) cross-lagged model (31). If such a model had shown significant cross-lagged correlations, this would mean that individual-level variation in one factor predicted individual-level variation in the other one while adjusting for autocorrelation. We did test such models, but significant cross-lagged correlations were not obtained.

For cross-lagged correlations in a Random Intercept Cross-Lagged model (RI-CLPM) or a Latent Curve Model with Structured Residuals to be significant, the statistical power would have to be sufficient. Factors like weak associations combined with a highly skewed “Crowded places” variable and moderate sample size may have contributed to the lack of significance in these models.

An overview of control measures which were implemented during the data collection period as well as number of reported cases of infection are shown in Supplementary Table S1. Of particular relevance for the present study are closures of cafés, pubs/bars, and restrictions on number of people at private gatherings which were all in force during the whole period of data collection. Prohibition of gatherings with more than 50 people were in force during Wave 1 only. The use of restrictive measures during the study period supports the relevance of our questions on visiting crowded places. We have, however, no reason to believe that variation in use of restrictions on behavior during the period of data collection has had systematic impact of patterns of associations between predictors and outcomes.

The data have several strengths that should be highlighted. The number of participants at Wave 1 was relatively high (*n* = 1,400), attrition was moderate (n at last data collection = 645), instruments for data collection were identical across waves, the same individuals were followed in up to six data collections, and data collections were carried out at regular intervals (every month). We have also analyzed the data with software ideal for examining processes of mutual influence between factors over time, and we have used maximum likelihood estimation, which is an adequate approach for data with some attrition, additional recruitment after Wave 1, and missing observations.

On the other hand, the instruments used for data collections are also associated with the critical limitation of failing to collect some potentially useful information. By following one of the most elaborated conceptual models available, such as the most recent version of Theory of Planned Behavior, we would have had a more complete and detailed coverage of the most important predictors. This may have contributed to strengthening the predictive power of the behavioral determinants.

Although we have characterized the level of attrition as moderate, attrition could still represent a source of bias in the present study. In addition, the proportion of invited people who decided to participate, was relatively low (Supplementary Figure S1). Responding repeatedly to questions about precautions taken to avoid transmission of infection is probably easer for those who comply with the authorities' recommendations. Low response rates and high attrition, however, tend to bias prevalence estimates more than associations between variables (36). To the extent that attrition contributes to reduced variation in outcomes or predictors, a general decline in association strength is likely to occur.

The survey was conducted *via* the internet, which could introduce a selection bias in terms of overrepresentation of skilled and frequent internet users. All data, including measurements of behavior, were self-recorded and therefore subject to recall bias.

Another possible problem is the way “Visited or intended to visit crowded places” has been measured. The number of times each of the venues were visited was not registered, only binary information about the kind of places visited. For example, a person who went to restaurants every day would obtain the same registration as a person who frequented restaurants once a month. Quantifying visits to crowded places more adequately might have yielded richer data and potentially stronger predictive power (30).

The items used for measuring “Visited or intended to visit crowded places” could have been expanded to include more frequently visited venues, in addition to “Supermarket or other store for food.” In this way it could be investigated to what extent these venues would constitute a second factor in addition to places visited more seldomly.

On the predictor side, there is also room for improvements. The principle of compatibility between predictors and outcome variables, as described by Fishbein and Ajzen (10), could have been practiced more strictly and additional predictors, first of all “Intentions” and “Subjective norms” could have been added. There also seems to be a need to improve the items for measuring “Vulnerability.”

## Conclusion

Baseline correlation analyses showed the relevance of many items in all domains to predict visiting or intending to visit crowded locations, particularly indicators of Perceived response effectiveness, Self-efficacy, and Barriers. Through a series of cross-sectional regression analyses on data from each of the six waves, we identified “Response effectiveness of individual action,” “Self-efficacy with regard to avoiding people,” and “Barriers” as important and consistent predictors of behavior. In a situation where all the cognitive predictors were highly correlated, these were the most powerful ones. Cross-lagged model analyses confirmed that cognitive factors predict social distancing behavior prospectively as well as vice versa, although associations were weak.

Our findings are consistent with the assumption that behavior may be influenced through health education and cognition-based behavior change approaches and suggests some potential to reduce people's visits to crowded locations during a pandemic.

## Data availability statement

The dataset analyzed in the study contains anonymized individual-level data. All data are stored securely, and confidentiality is protected in accordance with the Norwegian Data Protection Act, the General Data Protection Regulation of the European Union (GDPR), and in accordance with requirements of the Norwegian Health Research Act. Data will be made available to other researchers with fair requests after completion of our planned analyses.

## Ethics statement

The study was approved by the Regional Ethical Committee West (reference number 128391). Participants were adults who participated voluntarily in the survey and gave informed consent before completing the first questionnaire. The sample was drawn from a panel established by the data collection company. All panel members had already given their consent to be included in the internet panel and to be approached for online research.

## Author contributions

LV, BD, and BR in collaboration with members of the international CoMix team planned the study, supervised data collections, and were responsible for data facilitation. BR was PI for the Norwegian CoMix team. LA, ØV, and OS were responsible for data analyses and interpretation of results. LA drafted a first manuscript and was responsible for revisions. All authors examined the manuscript and provided suggestions and feedback. All authors contributed to the article and approved the submitted version.

## Supplementary material

Supplementary Tables S1–S3 Supplementary tables to Aarø et al.: Visiting crowded places.

Supplementary Figure S1 Recruitment and data collection timeline for the CoMix study, Norway.
