# Supplementary material for: Visiting crowded places during the COVID-19 pandemic. A panel study among adult Norwegians
Source: Front Public Health. 2022 Dec 15;10:1076090. doi: 10.3389/fpubh.2022.1076090 (PMC9797867; doi:10.3389/fpubh.2022.1076090)
Supplement: Supplementary Tables S1–S3 — Supplementary tables to Aarø et al.: Visiting crowded places. [file Data_Sheet_1.docx]

Table S1: Control measures implemented during the week of the CoMix data sampling (starting date of the data collection is indicated) and COVID-19 weekly incidence in Norway, April-September 2020.

|  | **Data collection period** | | | | | | **Dates during the study period that the measures were applied** |
| --- | --- | --- | --- | --- | --- | --- | --- |
| **Control measures** | **Wave 1,**  **24 April** | **Wave 2,**  **19 May** | **Wave 3,**  **9 June** | **Wave 4,**  **21 July** | **Wave 5,**  **25 Aug.** | **Wave 6,**  **23 Sept.** |  |
| Ban all events | Yes | No | No | No | No | No | 12/3/2020-6/5/2020 |
| Closure of kindergartens/daycare | No | No | No | Summer break | No | No | 12/3/2020-20/4/2020 |
| Closure of primary schools- | Yes until 26 April | No | No | Summer break | No | No | 12/3/2020-26/4/2020 |
| Closure of higher education/universities | Yes | Yes | Yes until 14th June | Summer break | No | No | 12/3/2020-14/6/2020 |
| Closure of secondary schools | Yes | No | No | Summer break | No | No | 12/3/2020-11/5/2020 |
| Closure of cafes-restaurants | Yes | Yes | No but with restrictions* | No but with restrictions* | No but with restrictions* | No but with restrictions* | 12/3/2020-31/5/2020,  Open but with restrictions* after 1/6/2020 |
| Closure of pubs/bars | Yes | Yes | No but restrictions applied* | No but with restrictions* | No but with restrictions* | No but with restrictions* | 12/3/2020-31/5/2020, Open but with restrictions* after 1/6/2020 |
| Closure of gyms, sports centres | Yes | No | No | No | No | No | 12/3/2020-14/5/2020 |
| Any gatherings above 50, indoors or outdoors not allowed | Yes | No | No | No | No | No | 7/5/2020-14/6/2020 |
| Teleworking strongly suggested | Yes | Yes | Yes | No | No | No | 10/3/2020-17/6/2021 |
| Private gathering restrictions | Yes  (Advice to not meet in groups >5) | Yes  (Advice to not meet in groups >20) | Yes  (Advice to not meet in groups >20) | Yes  (Advice to not meet in groups >20) | Yes  (Advice to not meet in groups >20) | Yes  (Advice to not meet in groups >20) | From 12/3 advised not to meet in groups of more than 5. Also advised to meet outdoors. From 7/5 advice to avoid gathering >20 people in private homes. |
| **Weekly Reported cases among all Norwegian population (sampling week)** | 359 (week 17) | 101 (week 21) | 80 (week 24) | 94 (week 30) | 374 (week 35) | 776 (week 39) |  |

Table S2: Meanscore descriptives after replacement of missing with means by wave

| Meanscore | Wave | Mean | SD | N | # of missing | N total |
| --- | --- | --- | --- | --- | --- | --- |
| “If I don’t follow government’s advice, I might spread the virus to others” (single item) | 1 | 4.42 | 1.04 | 1 366 | 34 | 1 400 |
|  | 2 | 4.33 | 1.05 | 1 165 | 17 | 1 182 |
|  | 3 | 4.32 | 1.06 | 1 001 | 11 | 1 012 |
|  | 4 | 4.46 | 0.94 | 914 | 17 | 931 |
|  | 5 | 4.45 | 0.96 | 749 | 19 | 768 |
|  | 6 | 4.43 | 0.92 | 635 | 10 | 645 |
| Response effectiveness –  Individual action | 1 | 3.52 | 0.49 | 1 375 | 15 | 1 400 |
|  | 2 | 3.46 | 0.48 | 1 173 | 9 | 1 182 |
|  | 3 | 3.46 | 0.51 | 1 005 | 7 | 1 012 |
|  | 4 | 3.48 | 0.47 | 925 | 6 | 931 |
|  | 5 | 3.47 | 0.48 | 759 | 9 | 768 |
|  | 6 | 3.49 | 0.50 | 637 | 8 | 645 |
| Response effectiveness – Individual action if someone in household has symptoms | 1 | 3.37 | 0.62 | 1 363 | 37 | 1 400 |
|  | 2 | 3.28 | 0.66 | 1 144 | 38 | 1 182 |
|  | 3 | 3.27 | 0.71 | 986 | 26 | 1 012 |
|  | 4 | 3.29 | 0.67 | 902 | 29 | 931 |
|  | 5 | 3.22 | 0.70 | 743 | 25 | 768 |
|  | 6 | 3.24 | 0.70 | 619 | 26 | 645 |
| Response effectiveness –  Restrictive measures | 1 | 3.32 | 0.53 | 1 378 | 22 | 1 400 |
|  | 2 | 3.18 | 0.55 | 1 162 | 20 | 1 182 |
|  | 3 | 3.19 | 0.56 | 994 | 18 | 1 012 |
|  | 4 | 3.25 | 0.53 | 911 | 20 | 931 |
|  | 5 | 3.16 | 0.53 | 756 | 12 | 768 |
|  | 6 | 3.16 | 0.53 | 628 | 17 | 645 |
| Self-efficacy – Avoiding people | 1 | 3.51 | 0.57 | 1 387 | 13 | 1 400 |
|  | 2 | 3.46 | 0.59 | 1 175 | 7 | 1 182 |
|  | 3 | 3.47 | 0.58 | 1 003 | 9 | 1 012 |
|  | 4 | 3.46 | 0.58 | 926 | 5 | 931 |
|  | 5 | 3.45 | 0.62 | 760 | 8 | 768 |
|  | 6 | 3.45 | 0.60 | 636 | 9 | 645 |

Table S2 (continued): Meanscore descriptives after replacement of missing with means by wave

| Meanscore | Wave | Mean | SD | N | # of missing | N total |
| --- | --- | --- | --- | --- | --- | --- |
| Self-efficacy – Stay home | 1 | 3.42 | 0.61 | 1 387 | 13 | 1 400 |
|  | 2 | 3.37 | 0.64 | 1 172 | 10 | 1 182 |
|  | 3 | 3.39 | 0.66 | 1 005 | 7 | 1 012 |
|  | 4 | 3.35 | 0.65 | 922 | 9 | 931 |
|  | 5 | 3.35 | 0.63 | 758 | 10 | 768 |
|  | 6 | 3.34 | 0.64 | 638 | 7 | 645 |
| Facilitating factors ¤ | 1 | 3.91 | 1.16 | 1 387 | 13 | 1 400 |
|  | 2 | 3.88 | 1.14 | 1 172 | 10 | 1 182 |
|  | 3 | 3.90 | 1.10 | 1 008 | 4 | 1 012 |
|  | 4 | 3.88 | 1.18 | 925 | 6 | 931 |
|  | 5 | 3.92 | 1.12 | 758 | 10 | 768 |
|  | 6 | 3.93 | 1.11 | 634 | 11 | 645 |
| Barriers | 1 | 2.25 | 1.16 | 1 375 | 25 | 1 400 |
|  | 2 | 2.26 | 1.14 | 1 167 | 15 | 1 182 |
|  | 3 | 2.32 | 1.15 | 998 | 14 | 1 012 |
|  | 4 | 2.29 | 1.15 | 920 | 11 | 931 |
|  | 5 | 2.37 | 1.17 | 751 | 17 | 768 |
|  | 6 | 2.37 | 1.18 | 635 | 10 | 645 |

¤ Two out of three items: If I could not work because of coronavirus, I would still get paid; I have enough food and supplies to last for 7 days, if I had to isolate myself

Table S3: Visited or intended to visit supermarket or other store for food by cognitive predictors and facilitating factors and barriers. Multiple binary logistic regression model, one for each Wave. Standardized predictors.

| Omnibus test of model coefficients: |  | Wave 1  (n=1400)  𝞦^2^=36.179; d.f.=8: p<.001) | Wave2  (n=1182)  𝞦^2^=25.515; d.f.=8: p<.001) | Wave3  (n=1012)  𝞦^2^=36.111; d.f.=8: p<.001) | Wave4  (n=931)  𝞦^2^=25.417; d.f.=8: p<.001) | Wave5  (n=768)  𝞦^2^=18.254; d.f.=8: p<.05) | Wave6  (n=645)  𝞦^2^=38.014; d.f.=8: p<.001) |
| --- | --- | --- | --- | --- | --- | --- | --- |
| Risk of spreading the coronavirus to others |  | 1.294*** | 1.315*** | 1.447*** | 1.350** | 1.357** | 1.630*** |
| Response effectiveness | Taking individual action | 1.333* | 1.127 | 1.297+ | 1.374+ | 1.281 | 0.943 |
|  | Taking individual action if someone in household has symptoms (ZIndAct2X) | 0.774* | 0.805 | .875 | 0.875 | 0.711+ | 0.740 |
|  | Restrictive measures | 0.886 | 0.796+ | .830 | 0.678* | 0.701* | 0.660* |
| Self-efficacy | Avoiding people | 0.941 | 1.095 | 1.046 | 1.081 | 1.003 | 0.944 |
|  | Staying home | 0.835 | 0.960 | .795 | .908 | 1.146 | 1.051 |
| Facilitating factors and barriers | Facilitating factors | 0.864+ | 1.082 | 1.024 | 1.204+ | 1.039 | 0.952 |
|  | Barriers | 0.873+ | 0.841+ | 0.790* | .795* | .901 | 0.846 |
