# Supplementary figures and images for: Visiting crowded places during the COVID-19 pandemic. A panel study among adult Norwegians
Source: Front Public Health. 2022 Dec 15;10:1076090. doi: 10.3389/fpubh.2022.1076090 (PMC9797867; doi:10.3389/fpubh.2022.1076090)

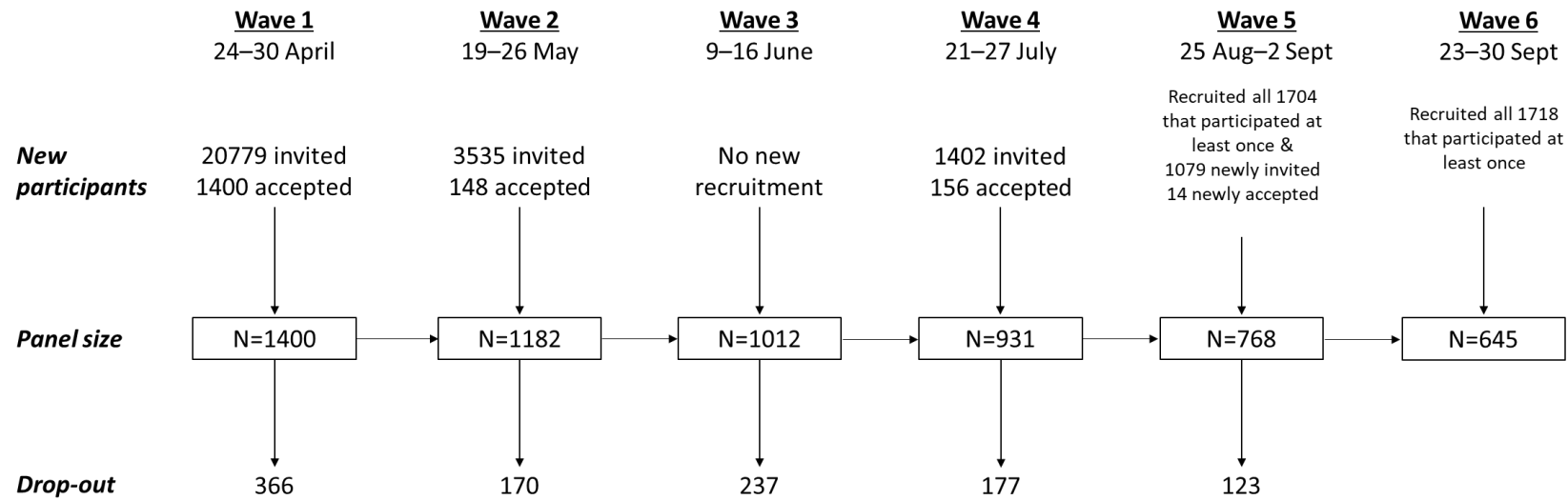

Fig. S1: Recruitment and data collection timeline, CoMix study, April to September 2020, Norway.

Supplement: Supplementary Figure S1 — Recruitment and data collection timeline for the CoMix study, Norway. [file Image_1.pdf]
